# The Impact of Estrogen and Estrogen-Like Molecules in Neurogenesis and Neurodegeneration: Beneficial or Harmful?

**DOI:** 10.3389/fncel.2021.636176

**Published:** 2021-03-08

**Authors:** Felipe A. Bustamante-Barrientos, Maxs Méndez-Ruette, Alexander Ortloff, Patricia Luz-Crawford, Francisco J. Rivera, Carlos D. Figueroa, Luis Molina, Luis Federico Bátiz

**Affiliations:** ^1^Immunology Program, Centro de Investigación e Innovación Biomédica (CiiB), Universidad de los Andes, Santiago, Chile; ^2^Cells for Cells, Santiago, Chile; ^3^Neuroscience Program, Centro de Investigación e Innovación Biomédica (CiiB), Universidad de los Andes, Santiago, Chile; ^4^Departamento de Ciencias Veterinarias y Salud Pública, Facultad de Recursos Naturales, Universidad Católica de Temuco, Temuco, Chile; ^5^Facultad de Medicina, School of Medicine, Universidad de los Andes, Santiago, Chile; ^6^Laboratory of Stem Cells and Neuroregeneration, Faculty of Medicine, Institute of Anatomy, Histology and Pathology, Universidad Austral de Chile, Valdivia, Chile; ^7^Center for Interdisciplinary Studies on the Nervous System (CISNe), Universidad Austral de Chile, Valdivia, Chile; ^8^Institute of Molecular Regenerative Medicine, Paracelsus Medical University, Salzburg, Austria; ^9^Spinal Cord Injury and Tissue Regeneration Center Salzburg (SCI-TReCS), Paracelsus Medical University, Salzburg, Austria; ^10^Laboratory of Cellular Pathology, Institute of Anatomy, Histology and Pathology, Facultad de Medicina, Universidad Austral de Chile, Valdivia, Chile; ^11^Facultad de Medicina y Ciencia, Universidad San Sebastián, Puerto Montt, Chile

**Keywords:** ERα/β, GPER1/GPR30, 17β-estradiol, bisphenol A, Alzheimer’s disease, Parkinson’s disease, hormone replacement therapy, neural stem/progenitor cells

## Abstract

Estrogens and estrogen-like molecules can modify the biology of several cell types. Estrogen receptors alpha (ERα) and beta (ERβ) belong to the so-called classical family of estrogen receptors, while the G protein-coupled estrogen receptor 1 (GPER-1) represents a non-classical estrogen receptor mainly located in the plasma membrane. As estrogen receptors are ubiquitously distributed, they can modulate cell proliferation, differentiation, and survival in several tissues and organs, including the central nervous system (CNS). Estrogens can exert neuroprotective roles by acting as anti-oxidants, promoting DNA repair, inducing the expression of growth factors, and modulating cerebral blood flow. Additionally, estrogen-dependent signaling pathways are involved in regulating the balance between proliferation and differentiation of neural stem/progenitor cells (NSPCs), thus influencing neurogenic processes. Since several estrogen-based therapies are used nowadays and estrogen-like molecules, including phytoestrogens and xenoestrogens, are omnipresent in our environment, estrogen-dependent changes in cell biology and tissue homeostasis have gained attention in human health and disease. This article provides a comprehensive literature review on the current knowledge of estrogen and estrogen-like molecules and their impact on cell survival and neurodegeneration, as well as their role in NSPCs proliferation/differentiation balance and neurogenesis.

## Introduction

Estrogens are cholesterol-derived sex hormones that play a fundamental role in many physiological processes (Kaludjerovic and Ward, 2012). The three main forms of estrogens: estrone, estradiol, and estriol, are produced endogenously by different organs and tissues, such as ovaries, testis, adipose tissue, and adrenal cortex. Estrogens are also used as part of pharmacological compounds in contraceptive therapies for pre-menopausal women and hormonal replacement therapies for post-menopausal women. These molecules have a strong affinity for the estrogen receptors (ERs) ERα, ERβ, and G-protein coupled ER1 (GPER1), through which, after binding, induce a direct or indirect genomic signaling to change the expression pattern of several genes (Fuentes and Silveyra, 2019). Additionally, some exogenous estrogen-like molecules, such as phytoestrogens and xenoestrogens, also have ER-binding capabilities (Nadal et al., 2018). By binding to these receptors, estrogens and estrogen-like molecules modulate cell proliferation (Gompel et al., 2004; Pisano et al., 2017; Molina et al., 2020), differentiation (Bassler, 1970; Grubbs et al., 1985; Imanishi et al., 2005), and survival (Weldon et al., 2004; Kishi et al., 2005; Yu et al., 2012) processes, by different downstream pathways. Furthermore, since estrogen receptors are ubiquitously located, estrogen-mediated signals modulate the biology of several tissues and organs, including the central nervous system.

Estrogens play a pivotal role in the adult and developing brain (Brannvall et al., 2002; Morissette et al., 2008), exerting neuroprotective effects under homeostatic and pathologic conditions. In that sense, estrogens can enhance anti-oxidant activity (Bellanti et al., 2013; Rekkas et al., 2014) and induce DNA repair (Bethea et al., 2016), two processes required to modulate the adverse effects of oxidative stress in the cell. Moreover, estrogens increase the expression of different growth factors and anti-apoptotic molecules, promoting cell survival and reducing neurodegenerative processes (Singh et al., 1995; Solum and Handa, 2002). They also positively modulate dendritic spines and axonal growth, synaptic transmission, and plasticity, processes required to maintain and enhance cognitive and memory performance (Wallace et al., 2006; Luine and Frankfurt, 2013). Neurodegenerative diseases are characterized by increased oxidative stress (Starkov, 2008), cell death, and concomitant loss of synapses; in this context, estrogen supplementation can prevent or reduce these negative features. Several studies have shown the beneficial effects of estrogens in reducing the progression and ameliorating the symptoms of Alzheimer’s and Parkinson’s disease (Kumar et al., 2010; Vail and Roepke, 2019).

Besides, estrogens are essential in maintaining the balance between proliferation and differentiation of embryonic and adult neural stem/progenitor cells (NSPCs), thus modulating neurogenic processes. Interestingly, classical (ERα and ERβ) and non-classical (GPER-1) estrogen receptors are expressed by cortical and hippocampal NSPCs (Brannvall et al., 2002; Waldron et al., 2010; Zhang et al., 2019; Zhong et al., 2019), and also by oligodendrocyte progenitor cells (OPCs) and post-mitotic oligodendrocytes, playing a central role in oligodendrogenesis and myelination (Kumar et al., 2010; Okada et al., 2010).

Here, we review the current knowledge on the role of estrogens and estrogen-like molecules in the neuro/gliogenic processes and the regulation of neuronal survival and neurodegeneration.

## Estrogens: Endogenous, Hormone-Based Therapies, and Estrogen-Like Molecules

**FIGURE 1:**
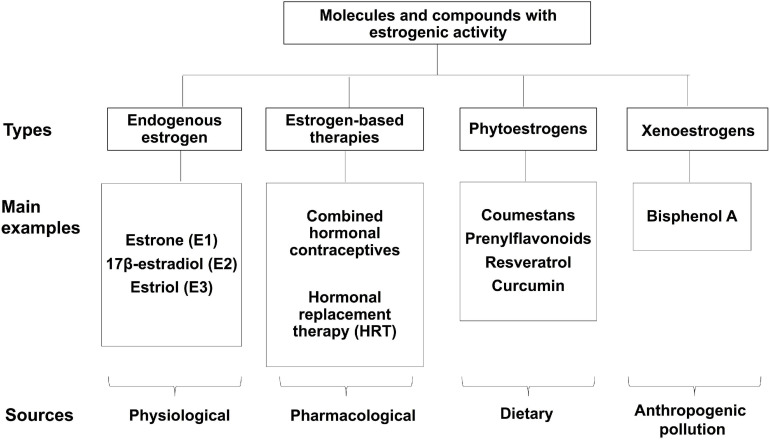
Molecules with estrogenic activity. Classification of the various molecules and compounds that display estrogenic activity.

Figure 1 summarizes main molecules and compounds with estrogenic activity, including endogenous estrogens, hormone-based therapies, and estrogen-like molecules.

### Endogenous Estrogens

Estrogens are not only involved in gender-specific processes such as development and maintenance of the female phenotype, germ cell maturation, and pregnancy (Yasar et al., 2017). They also play important roles in many other (non-gender-specific) physiological processes, including cardiovascular and nervous systems function (Auger et al., 2000; Denley et al., 2018). In that regard, Denley et al. (2018) have recently published that several human brain regions exhibit sexual dimorphism due to estrogenic influence, highlighting the role of estrogens in the differential adaptation of men and women’s brains. Thus, estrogens appear to play a key role in brain growth and maturation, opening novel perspectives on the effects of these hormones in brain homeostasis.

The two major biologically active estrogens in non-pregnant humans are estrone (E1) and estradiol (E2). E1 is considered to be a weaker form of estrogen and is the major estrogen found in women who are naturally menopausal. A third bioactive estrogen, estriol (E3), is the main pregnancy estrogen, but plays no significant role in non-pregnant women or men (Molina et al., 2017). Estrogens are produced through metabolic steroid pathways in ovaries, the adrenal cortex, adipose tissue, testes and the placenta (Miettinen et al., 2000; Pentikäinen et al., 2000; Kaludjerovic and Ward, 2012). E3 is produced primarily from 16α-hydroxy-dehydroepiandrosterone (DHEA) sulfate, 17β-estradiol or E2 is formed from aromatization of testosterone, whereas E1 is derived from aromatization of androstenedione (Yaghjyan and Colditz, 2011).

Estrogens are secreted cyclically, inducing a negative feedback on gonadotropins and modulating the hypothalamic-pituitary-gonadal axis (Jensen et al., 2010). Estradiol has important effects on the female reproductive organs, including breasts, uterus, and also the endometrium during the regulation of the menstrual cycle, as well as in non-reproductive tissues, such as skeletal, cardiovascular and nervous system. In men, estrogens increase serum levels of HDL (high-density lipoprotein) cholesterol, improving cardiovascular condition, and also contribute to the maintenance of bone mass and the maturation of sperm (Pentikäinen et al., 2000). Estradiol has been related to the modulation of appetite and the balance of glycemia, having a crucial role in the maintenance of the metabolic homeostasis and body weight (Rubinow, 2017). On the other hand, evidence indicates that estradiol also plays an important role in differentiating the human brain during early prenatal stages and even after birth (Gillies and McArthur, 2010). In fact, estrogens are fundamental in the generation of social behaviors related to sex.

The disruption of estrogens’ homeostasis is involved in the development or progression of a series of pathologies, which include several types of cancer (breast, ovary, colorectal, prostate, endometrium), osteoporosis, autoimmune diseases, cardiovascular diseases, obesity and/or insulin resistance (Cui et al., 2013; Patel et al., 2018), as well as neurodegenerative diseases (Gillies and McArthur, 2010; Cui et al., 2013; Villa et al., 2016) and mood disorders (Payne, 2003).

### Combined Hormonal Contraceptives and Hormone Replacement Therapy

Currently, the applications of sex hormone and gonadotropin therapies are rather broad and versatile, being used in several cases of female and male infertility (Lunenfeld et al., 2019; Casarini et al., 2020). On the other hand, the most extensive uses of estrogens correspond to the modulation of reproductive capacity by hormonal contraceptives, and hormone replacement therapy (HRT), aiming to reverse the estrogen deficit during post-menopause.

#### Hormonal Contraception

In women, most contraceptive therapies are based on the release of one or more sex hormones, usually containing a dose of synthetic progesterone (progestin), or a combination of progestin and synthetic estrogens (Wright et al., 2020). To improve their efficacy and tolerance, hormonal contraceptives have suffered many changes in their composition and dosage, reducing adverse reactions such as nausea, vomiting, and headache (Wright et al., 2020). In addition to avoiding unwanted pregnancies, hormonal contraceptives are also indicated in cases of menorrhagia, dysmenorrhea, acne, hirsutism, iron deficiency anemia, and endometriosis (Schindler, 2010). It also significantly impacts reducing the risk of endometrial, ovarian, and colon cancer (Schindler, 2010; Marsden, 2017).

The general mechanism of action of combined hormonal contraceptives (estrogens + progestogens) is based on the negative feedback that estrogens induce on the hypothalamus, inhibiting the secretion of gonadotropin release stimulating hormone (GnRH) and, consequently, preventing the increase in the secretion of gonadotrophins by the pituitary to stimulate ovulation in the middle of the cycle. In turn, progestogens inhibit follicular development and exert an anovulatory effect. In addition, the endometrium thins, and the cervical mucus becomes thicker and more impenetrable for sperm (Marsden, 2017; Regidor, 2018; Wright et al., 2020).

#### Hormone Replacement Therapy (HRT)

A growing number of studies indicate that estrogen therapy has a broad spectrum of beneficial effects in post-menopausal women (Behl and Lezoualc’h, 1998; Cacciatore et al., 1998; Zhao and Brinton, 2006; Robb and Stuart, 2010; Engler-Chiurazzi et al., 2017). Regarding this, menopause can be understood as a physiological event in women characterized by the end of menstrual cycles and the depletion of ovarian follicles (Santoro and Randolph, 2011). Hypoestrogenism, particularly of estriol, generates a group of symptoms known as the climacteric, which are characterized by vasomotor alterations, urogenital atrophy, and a series of metabolic changes, among other symptoms, which directly affect women’s quality of life (Santoro and Randolph, 2011; Blumel and Arteaga, 2018).

The higher life expectancies in the current population imply that more than half of women’s life passes in the post-menopausal stage. During this period, the morbidity and mortality patterns change, with chronic diseases becoming more relevant, such as malignant neoplasms, atherosclerosis, osteoporosis, arteriopathies, and neurodegenerative diseases (Blumel and Arteaga, 2018). Consequently, HRT aims to reduce the climacteric symptoms and the incidence of chronic diseases during this phase of women’s life.

The use of HRT dates back to the 1960s; however, after the appearance of prospective randomized studies that indicated an increase in the incidence of breast cancer, the establishment of this therapy has been adjusted to the presence of certain risk factors, symptoms, and the evolution of patients (Davey, 2018; Cagnacci and Venier, 2019). Nowadays, HRT supplements women with estrogens (mainly estradiol) or estrogens and gestagens during the menopausal transition. Although three types of estrogens are available pharmacologically—natural, equine, and synthetic—, natural and equine estrogens represent the most used in HRT (Bhavnani, 2003; Zhao and Brinton, 2006).

### Estrogen-Like Molecules: Phytoestrogens and Xenoestrogens

The study of estrogen-like molecules whose structure and effects are similar to those induced by estrogens has acquired increasing relevance in animals and humans (Molina et al., 2018). Phytoestrogens are plant-derived molecules with several metabolic and endocrine effects similar to those induced by estrogens. There are more than 4,000 phytoestrogens described, which can be divided into two chemical groups: flavonoids (coumestans and prenylflavonoids) and non-flavonoids (reviewed in Molina et al., 2018). By contrast, xenoestrogens are chemicals that act as potent endocrine disruptors by binding with a high affinity to classical and non-classical ERs, like bisphenol-A and bisphenol-S (reviewed in Nadal et al., 2018). Bisphenol-A (BPA) is the principal industrial compound used in the production of polymers of plastic polycarbonate. Bisphenol-A molecules are joined by ester bonds, which are disrupted by high temperatures or pH changes, generating their release into environments that commonly include mineral water bottles and lacquer coating in food cans (Brotons et al., 1995; Wagner and Oehlmann, 2009). Since phytoestrogens and xenoestrogens can be considered as omnipresent molecules in our daily diet, they are becoming an increasingly attractive focus of research on human health and disease (Table 1).

**TABLE 1:** Molecular and biological effects of endogenous estrogen and estrogen-like molecules in NSPCs.

**Ligands**	**Molecular findings**	**Biological effects**	**Model**	**References**
			**(E/P)**	
17β-estradiol	Activation of ERK1/2 and PI3K/Akt/mTOR	Improves proliferative activity and promotes oligodendrogenesis	Human-derived NSPCs (E)	Wang et al., 2008; Okada et al., 2010; Kumar et al., 2013
			Rat-derived NSPCs (E)	
			Mouse brain tissue (P)	
Tamoxifen, 4-OHTMX and endoxifen	Via GPER-1 in a PKCα independent-manner	Promote oligodendrogenesis	Rat-derived NSPCs (P)	Barratt et al., 2016; Gonzalez et al., 2016
			Rat brain tissue (P)	
			Rat glial precursor cells (P)	
Diarylpropionitrile	Activation of PI3K/Akt/mTOR pathway	Promotes oligodendrogenesis and maturation of OLs	Mouse corpus callosum and spinal cord (P)	Crawford et al., 2010; Khalaj et al., 2016
Fulvestrans (ICI-182,780)	N.D.	Suppresses proliferative activity	Rat-derived NSPCs (E)	Okada et al., 2010; Barratt et al., 2016
			Rat glial precursor cells (P)	
Hops-derived prenylflavonoids	N.D.	Promote neurogenesis and neurite outgrowth	Rat-derived NSPCs (P)	Oberbauer et al., 2013
			Dorsal root	
			ganglion neurons (E)	
Resveratrol	Activation of PKA-GSK3β-β.catenin, PKA-ERK1/2, p38 kinases and SIRT1 pathways	Improves proliferative activity and promote neurogenesis	Human cord blood-derived mesenchymal stem cells (P)	Kumar et al., 2016; Jahan et al., 2018
			Rat brain tissue (P)	
			Rat-derived NSPCs (E)	
	Activation of AMPK pathway	Suppresses proliferation and neurogenesis	Mouse brain tissue (P)	Tiwari et al., 2015b
			Mouse-derived NSPCs (E/P)	
Curcumin	Activation of Wnt pathway	Improves proliferative activity and promotes neurogenesis	Rat brain tissue (P)	Tiwari et al., 2016
			Rat-derived NSPCs (E)	
Bisphenol-A	Deregulation of Wnt pathway	Reduced proliferative activity and neuro/oligodendrogenesis	Rat brain tissue (P)	Tiwari et al., 2015a, b, 2016
			Rat-derived NSPCs (E)	

*NSPCs, neural stem/progenitor cells; 4-OHTMX, 4-hydroxytamoxifen; E, embryonic; P, postnatal; N.D., non-described.*

### Estrogen-Dependent Signaling Pathways and Cellular Effects

#### Classical Estrogen Receptors

In the 1960s, the group of Jensen and coworkers detected an intracellular protein in ovarian tissue able to interact with E2: the estrogen receptor α (ERα) (Jensen et al., 2010). A decade later, experiments performed by Kuiper et al. (1996) on mouse prostatic tissue showed another molecule able to link estrogen, named estrogen receptor β (ERβ) or ERβ1. Both proteins belong to the superfamily of nuclear receptors, which translocate from the cytosol to the nucleus in response to estrogens, and are widely known as “classical estrogen receptors” because they were the first receptors discovered to coupling to estrogen. Both receptors share a typical structure consisting of a carboxy-terminal ligand-binding domain, a DNA-binding site located in the center region, and an amino-terminal domain. The ligand-binding domain and DNA-binding site regions are highly conserved among these receptors, whereas the amino-terminal domain region has more significant variability on its sequence and length (Higa and Fell, 2013).

**FIGURE 2:**
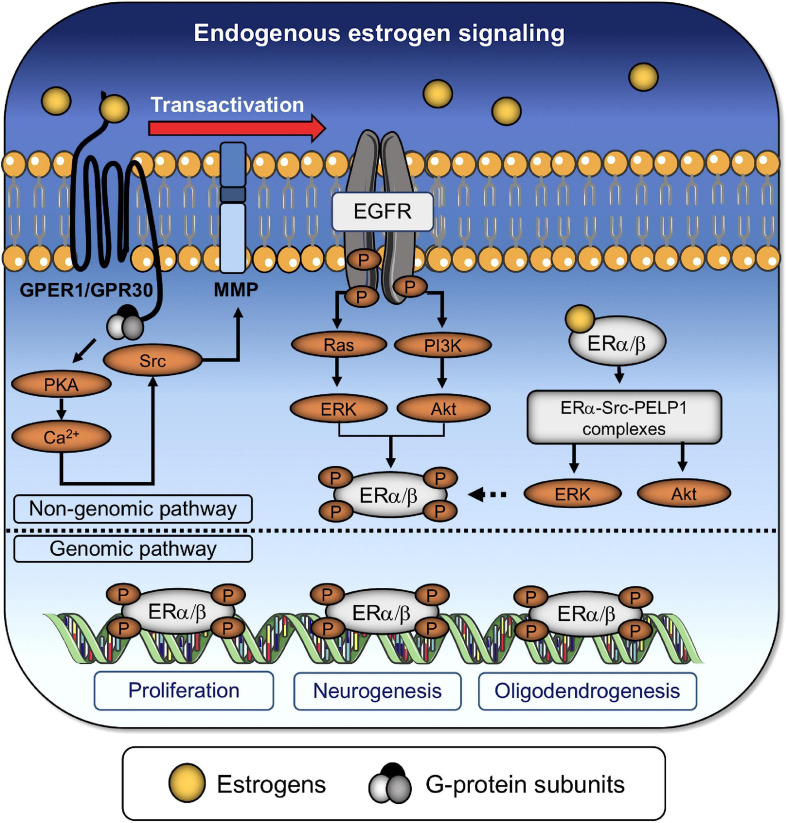
Endogenous estrogen regulates the proliferation and differentiation of NSPCs. Endogenous estrogen binds to both classical and non-classical ERs. Transactivation of the epidermal growth factor receptor (EGFR) via GPER-1 promotes the formation of dimers or heterodimers between nuclear receptors through (i) the activation of protein kinase A (PKA), (ii) an increase in the intracellular Ca^2+^ concentration, and (iii) the subsequent activation of the non-receptor tyrosine kinase (Src) and matrix metalloproteinases (MMP). EGFR promotes the downstream activation of the small GTPase (RAS)/phosphatidylinositol 3-kinase (PI3K) and the extracellular-signal-related kinase (ERK)/protein kinase B (also known as Akt) signaling pathways, which, in turn, phosphorylate of nuclear receptors, denoting the end of the non-genomic pathway. Alternatively, estrogens can directly bind to dimerized nuclear receptors (ERα/β) to activate the ERK/Akt pathways and promote their own phosphorylation. The genomic pathway involves the translocation of phosphorylated ER dimers into the nucleus for controlling the expression of genes associated with cell survival/proliferation, neurogenesis, and oligodendrogenesis.

**FIGURE 3:**
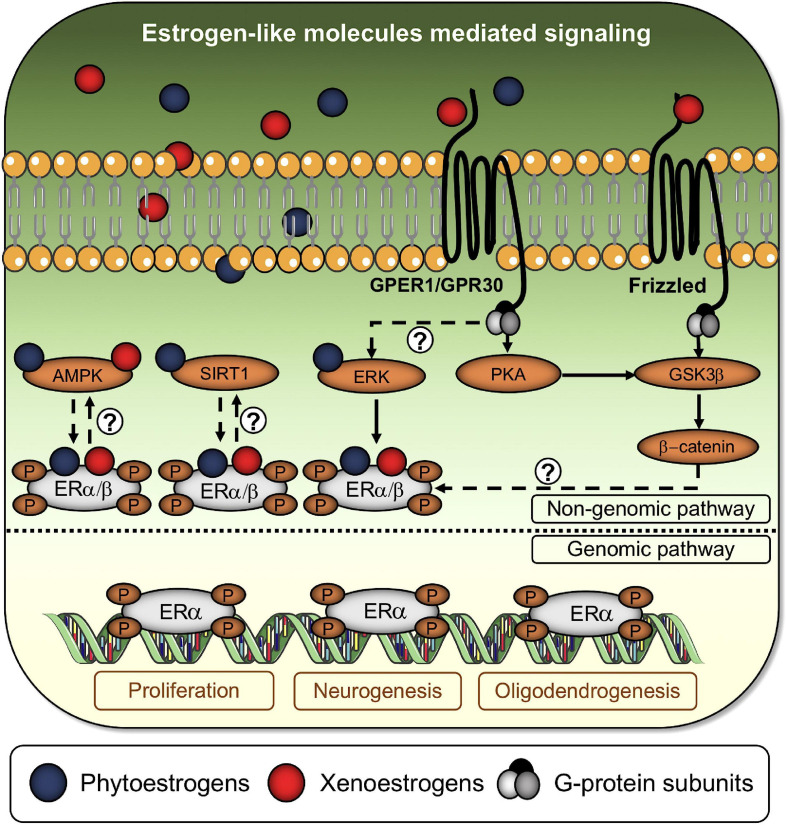
Estrogen-like molecules activate the classical estrogen signaling cascade and alternative pathways. Phytoestrogens and xenoestrogens bind to classical and non-classical ERs to enhance or disrupt estrogenic activity. Alternatively, xenoestrogens are capable of promoting ER dimers phosphorylation through the Frizzled/GSK3β/β-catenin signaling axis. Question marks denote the absence of a well-established axis between phosphorylated ERs dimers and soluble AMP-activated protein kinase (AMPK), sirtuin-1 (SIRT1), and extracellular-signal-related kinase (ERK). Once phosphorylated, ERs dimers translocate to the nucleus to begin the genomic pathway, controlling the expression of genes associated with cell survival/proliferation, neurogenesis, and oligodendrogenesis.

Both receptors display a differential expression in normal tissues. The ERα is mainly expressed in female reproductive tissues, kidney, liver, bone, and white adipose tissue (Kuiper et al., 1996). By contrast, the ERβ is expressed in the ovary, lung, prostate, colon, kidney, immune cells, and cardiovascular tissues. Both receptors are also present in the nervous system, being ERβ more abundantly expressed than ERα (Kuiper et al., 1996). Most evidence supports that ERα regulates proliferation and survival, whereas ERβ modulates ERα when co-expressed (Higa and Fell, 2013). These receptors form homo- and heterodimers that subsequently translocate into the nucleus (Figures 2, 3). This process is known as genomic or nuclear response and entails up- and down-regulation of diverse genes associated with cell proliferation, survival, and migration (Musgrove and Sutherland, 2009; Haldosen et al., 2014). On the other hand, the concept of non-genomic or cytoplasmic response was introduced in 1967, when Szego and Davis observed an increase in cyclic adenosine monophosphate (cAMP) in the MCF-7 breast cancer cell line pretreated with estrogens (Szego and Davis, 1967). More recently, it has also been reported that several cell types and tissues show different non-genomic responses (Molina et al., 2017, 2018). For example, primate-derived neurons and neurons derived from human NSPCs exhibit rapid oscillation in the intracellular calcium concentration after being stimulated with estrogens or ERβ selective agonists (Zhang et al., 2010; Kenealy and Terasawa, 2011). Interestingly, the exhaustive analysis of these responses has helped to decipher alternative pathways that are not regulated by classical estrogen receptors and are triggered by estrogen mimetics. In this scenario have appeared the so-called non-classical estrogen receptors as we described next.

#### GPR30/GPER-1: A Non-classical Estrogen Receptor

Initially, GPR30 (G protein-coupled receptor 30) was considered an “orphan receptor” (Filardo et al., 2000). However, once its ability to interact with high affinity with E2 was determined, GPR30 was renamed GPER-1 (G protein-coupled estrogen receptor 1) (Revankar et al., 2005). GPER-1 belongs to the family of G protein-coupled receptors, and, in humans, its gene is located in chromosome 7p22.3, which encodes a sequence of 375 amino acids with a relative molecular mass of 41 kDa. As with other G protein-coupled receptors, GPER-1 is a seven-transmembrane domains receptor located at the plasma membrane, with its amino and carboxyl groups oriented to the extra- and intracellular environments, respectively (Figures 2, 3; Molina et al., 2017, 2018). When GPER-1 couples to estrogen, rapid and transient activation of diverse signaling pathways takes place. Most of these responses depend on the transactivation of epidermal growth factor receptor (EGFR) and its downstream targets phosphoinositide 3-kinase (PI3K)/serine-threonine-specific protein kinase B (also known as Akt) and extracellular signal-regulated kinases 1/2 (ERK 1/2) signaling pathways (Figure 2; Filardo et al., 2000). The ubiquitous distribution of GPER-1 supports many key functions that have been attributed to this receptor. Many tissues, including hepatic, nervous and adipose tissues, as well as cells from the circulatory and immune systems, express GPER-1. Furthermore, it is also expressed in prostate stromal cells (Jia et al., 2016), breast cancer cell lines (Ahola et al., 2002), cancer stem and germ cells (Chieffi and Chieffi, 2013), and osteoblast progenitors (Teplyuk et al., 2008).

#### Role of Estrogens in Cell Proliferation, Differentiation and Survival

Estrogens are recognized as a significant steroidal mitogen for epithelial cells, commonly related to oncogenesis (Anderson et al., 1998). In this sense, experiments carried out by our group on breast cancer cell lines support that continuous pharmacological blockade of classical ERs leads to the overexpression of GPER-1 (non-classical ER) as an alternative pathway to promote proliferation (Molina et al., 2020). Also, studies carried out in cancer-associated fibroblasts derived from breast cancer patients show that the GPER-1/EGFR signaling axis increases the expression of several cell cycle regulatory genes (Pisano et al., 2017). Indeed, the mechanisms involved in E2-mediated proliferation are associated with the inhibition of the tumor suppressor protein, p53, which, in turn, generates the downstream activation of cyclin-dependent kinases (CDKs) and, consequently, the release of the retinoblastoma protein (Rb) that promote the progression of the cell cycle by activating diverse transcription factors (Gompel et al., 2004).

On the other hand, E2 is also involved in the differentiation of diverse types of normal tissues, being critical to the generation of lobular-alveolar cells and the development of galactophorous ducts (Bassler, 1970). Similarly, E2 is essential for the terminal differentiation of the mammary gland (Gompel et al., 2004); while its impact on the differentiation of epithelial cells was described early in the ’80s as a critical pathogenic mechanism underlying the development and growth of tumors (Grubbs et al., 1985). Additionally, it has been described that estrogens can regulate the differentiation of endothelial progenitor cells derived from bone marrow through mechanisms that include an increase in Akt phosphorylation and modulation of the telomerase activity, which consequently delays the onset of senescence (Imanishi et al., 2005).

Finally, the pro-survival effects of estrogens have been demonstrated in diverse cell types, including germ cells from human testes (Pentikäinen et al., 2000), breast cancer cells (Weldon et al., 2004; Yu et al., 2012), and neurons (Kishi et al., 2005). In germ cells, Pentikäinen et al. (2000) reported that classical ERs are found in early meiotic spermatocytes and elongating spermatids. Interestingly, relatively low concentrations of E2 are capable of robustly inhibiting germ cell apoptosis in cultured segments of human seminiferous tubules without survival factors (i.e., serum and hormones). Likewise, experiments performed by Yu et al. (2012) support that E2 regulates the expression of Bcl-2, an anti-apoptotic protein, and survivin, which inhibits caspase activation, by the suppression of different miRNAs, including miR-16, miR-143 and miR-203. Furthermore, the authors reported that the MCF-7 breast cancer cell line shows an E2-dependent increase in its survival rate, which is abrogated by pre-treating them with the anti-estrogenic compounds ICI-182, 780, or raloxifene (Yu et al., 2012). In turn, the over-expression of p-160 co-activators has been shown to enrich E2-mediated gene expression and improve cell survival by suppressing the tumor necrosis factor α (TNFα) activity (Yu et al., 2012), supporting the participation of estrogens in the maintenance of cell survival.

The data mentioned above demonstrates that estrogen-mediated signaling is crucial in the biology of different cell types, modifying their proliferative capacity, differentiation, and survival. In fact, as will be discussed in the next sections, NSPCs and neurons are also subjected to this type of signaling.

## Estrogens in Neurodegeneration

### Estrogens and Their Role in Cell (Neuronal) Survival: Neuroprotection

Several *in vitro* and *in vivo* studies indicate that estradiol, and metabolites derived from phytoestrogens such as trans-resveratrol, are capable of promoting survival in neurons subjected to diverse stress conditions (Behl and Lezoualc’h, 1998; Robb and Stuart, 2010; Choi et al., 2020). Some conjugated equine estrogens (i.e., Premarin) are widely used to reduce climacteric symptoms and have also been shown to promote increased neuronal functioning, counteracting aging-associated cognitive decline and preventing Alzheimer’s disease (Zhao and Brinton, 2006; Engler-Chiurazzi et al., 2017). Although the mechanisms by which HRT promotes neuroprotection are mostly unknown, it has been proposed that signaling pathways mediated by both classical estrogen receptors and GPER-1 are involved in the anti-oxidant and anti-inflammatory effects of estrogens in nervous tissue (Unfer et al., 2015; Vail and Roepke, 2019; Guo et al., 2020).

#### Anti-oxidant Activity

Various neurodegenerative diseases are characterized by decreased mitochondrial activity, reduced oxidative phosphorylation, and increased reactive oxygen species (ROS) production in the CNS (Starkov, 2008). Oxidative stress is also amplified by the loss of anti-oxidant capabilities and increased production of inflammatory cytokines; both processes are gradually extended by aging. In that sense, mitochondria are considered the primary producer of ROS (Murphy, 2009).

Estrogens can act as pro-oxidant or anti-oxidant agents depending on cell types and ratio of different types of estrogen receptors. In this context, estrogens can produce reactive oxygen species by increasing mitochondrial activity and redox cycling of estrogen metabolites (Kumar et al., 2010). On the other hand, estrogens’ phenolic hydroxyl group can act as an anti-oxidant agent, being a protective factor against cardiovascular and neurodegenerative diseases (Kumar et al., 2010). Clinical data have shown lower oxidant stress and better anti-oxidant activity in the brain of pre-menopausal women, when compared with same-age men and older women, indicating the neuroprotective role of ovarian hormones against oxidative stress (Bellanti et al., 2013; Rekkas et al., 2014). Thus, experiments with ovariectomized (OVX) rats have shown a reduction in the anti-oxidant activity of superoxide dismutase (SOD) in the hippocampus, a process that contrasts with an increase in the pro-oxidant enzyme monoamine oxidase (MAO) in the same region (Huang and Zhang, 2010). As expected, oxidative damage and mitochondrial dysfunction are more evident under this condition (Navarro et al., 2008). The effect of OVX over mitochondrial functions appears to be related to changes in the fatty acid profile of the mitochondria membrane, where cardiolipin is reduced and more exposed to peroxidation (Borras et al., 2003; Baeza et al., 2008; Claypool and Koehler, 2012). In addition, as dysfunctional mitochondrial pools need to be removed (by processes such as mitophagy) to reduce ROS levels, new mitochondria are required to maintain energy levels in the cell. In that sense, estrogen positively regulates the expression of proteins related to mitochondrial biogenesis, such as the nuclear respiratory factor-1 (NRF-1) and the peroxisome proliferator-activated receptor-gamma coactivator 1 (PGC-1) (Kemper et al., 2013; Klinge, 2017).

#### DNA Repair

When DNA is exposed to ROS, it induces an oxidative base modification that could result in transcriptional mutagenesis (Bregeon et al., 2009) or in DNA single-strand breaks (SSBs) (Lindahl, 1993), two processes that leads to genomic instability and cell death (Rodier et al., 2009). The base excision repair (BER) pathway is one of the principal contributors to DNA repair, as its impairment is positively associated with brain aging and age-associated neurodegenerative diseases (Zarate et al., 2017). BER is initiated by DNA glycosylases, such as NTH1 and 8-oxoguanine-DNA-glycosylase (Ogg1), which recognize and remove the specific inappropriate base to form basic (apurinic/apyrimidinic; AP) sites. These sites are cleaved by the AP endonuclease, APE1, and corresponding nucleotides are then located into the gap by a DNA polymerase through two different sub-pathways, to finally be ligated by a DNA ligase (Krokan and Bjoras, 2013). Interestingly, Ogg1 activity significantly decreases with age in neuronal extracts from rat brains, and APE1 is reduced in neurons and astrocytes from the frontal and parietal cortex of old rats (Kisby et al., 2010; Swain and Subba Rao, 2011). In this context, it has been shown that estrogen supplementation positively regulate the transcription of APE1 and NTH1 in the dorsal raphe of OVX old macaques (Bethea et al., 2016). Additionally, estrogen appears to enhance the transcription of DNA repair enzymes in the cerebral cortex after hypoxia, contributing to reducing oxidative stress (Dietrich et al., 2013).

#### Growth Factors

Neuroprotective roles of estrogens are closely related to the synthesis of growth factors. The brain-derived neurotrophic factor (BDNF) is a neurotrophin necessary for brain development, neurogenesis (Scharfman and MacLusky, 2006), neuronal survival, synaptic plasticity, and memory formation (Heldt et al., 2007; Bekinschtein et al., 2014). Estrogens can induce BDNF expression through direct binding to an estrogen-sensitive response element (ERE) in the BDNF gene (Scharfman and MacLusky, 2006). Indeed, hormone influence over BDNF expression has been demonstrated in gonadectomized female and male rats, showing decreased BDNF mRNA levels in the hippocampus that can be rescued after estradiol supplementation (Singh et al., 1995; Solum and Handa, 2002). Moreover, BDNF mRNA expression is also rescued in the neocortex and the olfactory bulb of the OVX rats by estrogen treatment (Sohrabji et al., 1995; Gibbs, 1999). At the same time, estrogen supplementation and BNDF upregulation promote the expression of other trophic factors such as the nerve growth factor (NGF) and the neuropeptide Y (NPY) (Barnea and Roberts, 2001; Nakamura and McEwen, 2005; Iughetti et al., 2018). Interestingly, NPY has been reported to increase neurogenesis in the dentate gyrus (Malva et al., 2012).

#### Synaptic Plasticity

Estrogens also have a neuroprotective role favoring synaptic formation, a process required for the survival of new neurons. Interestingly, estrogen receptors ERα and ERβ are located in the axons and synaptic terminals of pre-synaptic cells and also in the dendritic spines (structures crucial for synaptic transmission) of post-synaptic cells (McEwen and Milner, 2007), indicating a role in local regulation of synapsis instead of exerting an effect at nuclear transcription (Zarate et al., 2017). The importance of ovarian hormones for dendritic spines is evident since the number of dendritic spines in the hippocampal CA1 and medial prefrontal cortex are reduced in OVX rats (Wallace et al., 2006). Furthermore, this depletive process can be rescued after estrogen supplementation, increasing the number of spines (Luine and Frankfurt, 2013) and spine synapses in CA1 (Woolley and McEwen, 1992). Additionally, estrogens seem to be necessary for axonal growth and synaptic plasticity (Kretz et al., 2004; von Schassen et al., 2006), improving the performance on different memory and cognition tests (Wallace et al., 2006).

#### Cerebral Blood Flow

Another neuroprotective role of estrogens is closely related to their function as modulators of cerebral perfusion. By binding to ERα in endothelial cells, estrogens induce nitric oxide (NO) release, consequently producing vasodilation (Chambliss et al., 2000). Interestingly, post-menopausal women exhibit a progressive reduction in blood flow (Penotti et al., 1996), and the impedance of this flow appears to be reduced after starting hormone (estrogen) replacement therapy (HRT) (Cacciatore et al., 1998).

### Estrogens and Neurodegenerative Diseases

Experimental and clinical data suggest that estrogens have protective effects against neurodegenerative diseases (Kumar et al., 2010; Vail and Roepke, 2019). Indeed, women affected by premature menopause under 40 years of age and who did not receive estrogens treatment show an increased risk of developing cardiovascular and neurodegenerative disorders, which correlate with an increase in mortality (Shuster et al., 2010). A recent meta-analysis that included epidemiological studies regarding the effects of hormone replacement therapy (HRT) on Alzheimer’s and Parkinson’s disease suggests that this type of therapy acts as a highly beneficial factor for post-menopausal women (Song et al., 2020).

#### Alzheimer’s Disease (AD)

AD is a progressive neurodegenerative pathology that leads to irreparable loss of hippocampal cells, negatively affecting the cognition and memory of patients (Dubois et al., 2016). Interestingly, post-menopausal women have a higher prevalence of this pathology than men (Li and Singh, 2014; Zagni et al., 2016), and a faster cognition decline, indicating a possible role of sex hormones in the evolution of AD. In line with these findings, altered β-amyloid deposition can inhibit aromatase, a key enzyme for converting androgens into estrogens (McCullough et al., 2003; Overk et al., 2012). On the other hand, HRT lowers the risk for AD and also slows the decline in cognitive functions in patients with AD (Yaffe et al., 2000).

The positive effect of estrogens in AD can be explained through different contexts. AD is characterized by a progressive reduction in the num ber and maturation of adult neurons in the dentate gyrus (DG) of the hippocampus (Moreno-Jimenez et al., 2019), suggesting an impairment in hippocampal neurogenesis that is closely correlated with the loss of hippocampal functions and cognitive decline (Hollands et al., 2017; Morello et al., 2018). As neurogenesis seems to be crucial to maintain cognitive functions, it has been suggested that this process enhance memory and prevent cognitive impairment. In that sense, estrogen treatments appear to promote the number of proliferating cells and synaptic biomarkers in the hippocampus (Dorostkar et al., 2015), with an increment of NSPC proliferation in the DG (Fahnestock et al., 2002; Sopova et al., 2014). Moreover, estrogens induce NSPC proliferation under pathological conditions, reducing the levels of oxidative stress and apoptosis (Correia et al., 2010; Anastasio, 2013) and improving cognitive and memory performance (Vaucher et al., 2002; Shohayeb et al., 2018).

As in OVX or aged rats, mitochondrial dysfunction and overproduction of ROS are two common characteristics of AD animal models and patients with AD (Starkov, 2008). The anti-apoptotic effects of estrogens in AD are related to the positive modulation of the anti-apoptotic protein Bcl-2 and the negative regulation of the pro-apoptotic protein Bax (Green et al., 1997; Pike et al., 2009; Correia et al., 2010). In that sense, it has been recently reported that estrogens enhance the expression of the miR-125b, a miRNA that suppresses the expression of the pro-apoptotic genes, Bak1 and p53 (Micheli et al., 2016; Amakiri et al., 2019). Additionally, estrogen can enhance survival by activating the BDNF gene and its downstream pathways (Gibbs, 1998; Bhattacherjee et al., 2014), influencing processes such as neurite outgrowth and spinogenesis, which can finally enhance memory and cognitive performance.

#### Parkinson’s Disease (PD)

Clinical data show that women with low estrogen exposure can develop PD earlier than women with high estrogen exposure (Ragonese et al., 2004), indicating a role for this sex hormone in PD’s pathogenesis. In that sense, estrogens also appear to have a protective role in PD progression and treatment response (Saunders-Pullman et al., 1999; Shulman, 2002; Jin, 2020). Estrogens could positively affect dopamine neurotransmission by reducing the expression of catechol-O-methyltransferase (COMT), the enzyme responsible for dopamine degradation (Jiang et al., 2003). In that sense, HRT appears to potentiate the effect of the most common anti-parkinsonian drug, levodopa, reducing its response threshold and enhancing its sensitivity (Gomez-Mancilla and Bedard, 1992; Tsang et al., 2000; Yadav et al., 2017). Additionally, the protective effects of estrogens in PD are also related to the activation of the mitogen-activated protein kinase (MAPK) signaling pathway and the consequent upregulation of Bcl-2, as demonstrated in dopaminergic cells in mesencephalic slice cultures (Wang et al., 2011). Moreover, estrogen can also act on the PI3K/Akt pathway to induce the expression and upregulation of BDNF and dopamine transporter (DAT) in dopaminergic cells of the midbrain (Yi et al., 2016). As discussed previously, BDNF has a neuroprotective role, favoring survival and enhancing synaptic plasticity and transmission. Additionally, estrogen supplementation appears to have an effect against oxidative stress in PD brains (Numakawa et al., 2011; Jin, 2020).

#### Brain Ischemia and Stroke

Brain ischemia is a condition in which there is insufficient blood flow into the brain, leading to cerebral infarction and ischemic strokes. This situation leads to neurodegeneration (cell death) in infarcted regions and global brain inflammation (Radak et al., 2017). In that sense, estrogens can act as anti-inflammatory agents in the blood vessel wall, protecting it from cytokines and free radicals. Pretreatment with estrogen prevents blood vessel damage in animal models of brain ischemia (Rusa et al., 1999). Interestingly, these neuroprotective features have also been shown in humans, where post-menopausal women taking HRT, had fewer and smaller damaged areas after an ischemic event comparing to controls (non-HRT) (Gibson et al., 2006). These effects are mediated, in part, by the positive activation of the ERK/MAPK signaling pathway (Jover-Mengual et al., 2007).

## Estrogens and Estrogen-Like Molecules in Neurogenesis and Gliogenesis

### Neurogenic Niches: Adult Neural Stem/Progenitor Cells (NSPCs)

Stem cells are able to undergo proliferative divisions that produce additional stem cells with the same properties, and divisions that produce daughter cells that differentiate into multiple cell types (Homem et al., 2015). Stem cells can be “pluripotent” precursor cells that give rise to all cell types within an organism, or “multipotent” precursor cells that have more restricted potential, and differentiate into a subset of cell types. Neural stem cells (NSCs) are multipotent stem cells that can also be referred to as “progenitor” cells (Martinez-Cerdeno and Noctor, 2018). Thus, in this article we will refer to these cells as neural stem/progenitor cells (NSPCs), leaving the term of neural progenitor cells (NPCs) to those cells that are descendants of NSCs and have a restricted cell linage potential: neuronal progenitor cells, astrocyte progenitor cells, oligodendrocyte progenitor cells, etc.

Postnatal neurogenesis has been well demonstrated in two specific regions of the mammalian brain: the subventricular zone (SVZ) of the lateral ventricles and the subgranular zone (SGZ) of the dentate gyrus (DG) in the hippocampus (Gross, 2000; Kaplan, 2001). In both regions, NSPCs are immersed in a particular microenvironment composed of diverse cellular constituents, such as astrocytes, pericytes, ependymocytes, and vascular and microglial cells (Batiz et al., 2015; Bjornsson et al., 2015). Besides, it has been described other neurogenic niches able to generate new neurons and glial cells in response to brain injury or some other inductive stimuli. However, their magnitude and relevance remain still under discussion (Kokoeva et al., 2005; Cameron and Dayer, 2008; Lin and Iacovitti, 2015).

In the SVZ, type B cells—adult multipotent NSPCs— are located below ependymal and ependymal-like cells, which line the walls of the lateral ventricles (Mirzadeh et al., 2008). Ultrastructural and immunolabeling studies have revealed that type B cells display only a small fraction of apical surface and a single basal body. These cells contact the cerebrospinal fluid by short apical processes, and blood vessels by their long basal processes. Thus, the organization of these cells, i.e., ependymal, ependymal-like, and type B cells, confers a pinwheel architecture to the ventricular surface (Mirzadeh et al., 2008). Also, type B cells express diverse stem and glial markers (Batiz et al., 2015). As mentioned earlier, type B cells can divide and differentiate into type C cells, which represent a population of intermediate/transit-amplifying progenitor cells that, in turn, give rise to neuroblasts or type A cells (Doetsch et al., 1997; Parras et al., 2004; Merkle and Alvarez-Buylla, 2006). Finally, type A cells are guided to the olfactory bulb, where they gradually differentiate into two types of postmitotic GABAergic interneurons: the granular neurons and the periglomerular neurons (Gage, 2000; Kriegstein and Alvarez-Buylla, 2009; Ming and Song, 2011). Namely, the displacement of type A cells from the SVZ of the lateral ventricles to the olfactory bulb occurs through a tunnel-like structure composed of vascular cells and an astrocyte network driving the rostral flow of neuroblasts: the rostral migratory stream (Nguyen-Ba-Charvet et al., 2004).

In the SGZ, radial glia-like or type 1 cells—adult multipotent NSPCs— also exhibit a multistep differentiation process as described for the SVZ niche, beginning with the expansion of type 2a cells (transient amplifying progenitor cells) (Seri et al., 2001; Batiz et al., 2015). Then, type 2a cells divide into type 2b cells, which are more restricted intermediate progenitors, giving rise to type 3 cells (neuroblasts). Finally, type 3 cells divide into glutamatergic granule cells that are guided by astrocyte processes that function as instructive railways for their migration and subsequent integration into existing neuronal circuits in the granular cell layer (Seri et al., 2001; Aimone et al., 2014; Batiz et al., 2015). Of note, the processes of migration and integration of neurons in the hippocampus are remarkably shorter than through the rostral migratory stream (Shapiro et al., 2005).

### Estrogen Receptors in the Neurogenic Niches

In the two postnatal neurogenic niches described previously, the SVZ of the lateral ventricles and in the SGZ of the DG, multipotent NSPCs -type B and type 1 cells, respectively- express estrogen receptors. Interestingly, different studies have shown the expression of estrogen receptors in embryonic and adult NSPCs. Brannvall et al. (2002) demonstrated that ERα and ERβ are both expressed by cortical embryonic NSPCs obtained at early developmental stages. Interestingly, ERα levels are relatively high at embryonic day (E) 15 and decrease in later developmental stages. On the other hand, ERβ levels remain largely constant during development and are higher than ERα levels in cortical adult NSPCs (Brannvall et al., 2002).

Interestingly, Waldron et al. (2010) have shown that both ERa and ERb increase their levels in cortical adult NSPCs isolated from aged rats. Regarding the hippocampus, both ERα and ERβ have been observed in hippocampal NSPCs of human post-mortem samples and a human hippocampal progenitor cell line (Smeeth et al., 2020). Recent studies have shown that NSPCs obtained from Sprague Dawley rat embryos not only express ERα and ERβ, but also GPER-1 (Zhang et al., 2019). Besides multipotent NSPCs, Haumann et al. (2020) recently observed the expression of GPER1 in transient amplifying progenitor cells and neuroblasts from the SVZ of recently born and adult female rats.

### Biological Implications of Estrogens in Neurogenesis and Oligodendrogenesis

Experiments performed by Wang et al. (2008) using human-derived NSPCs revealed a significant increase in their proliferative activity after estrogen stimulation. In fact, 17β-estradiol (E2) induces time- and dose-dependent effects associated with an increase in DNA replication and up-regulation of cell cycle proteins. Remarkably, ERβ-, but not ERα-specific ligands, lead to increased phosphorylation of ERK 1/2, which initiates cell cycle entry (demonstrated by the up-regulation of mitotic markers such as CDK1/cdc2 and PCNA), DNA replication, and duplication of the centrosome -the major microtubule-organizing center in the cell- in dividing cells (Wang et al., 2008). Thus, in these cells, E2 effects appear to be predominantly mediated by ERβ. Similarly, primary cultures of embryonic rat-derived NSPCs respond to E2 increasing their proliferation (Brannvall et al., 2002).

GPER-1 has been found to be widely expressed in NSPCs (Zhang et al., 2019; Zhong et al., 2019). Functionally, NSPCs isolated from Sprague-Dawley rats, and treated with 10 nM E2 for 2 days, significantly increase their proliferative rate, while doses of 50 nM notably inhibit this process. Additionally, treatment with 10 nM E2, for 7 days, stimulated differentiation to the neuronal lineage, and inhibited differentiation to astrocytes (Zhang et al., 2019). However, the participation of GPER-1 in these cellular responses was not specifically verified. Recently, Zhong et al. (2019) stimulated mouse-derived NSPCs with different concentrations of G1, a selective GPER-1 agonist, showed a dose-dependent decreased proliferation, which was blocked by selectively inhibiting GPER-1.

It has also been demonstrated that ERs are pivotal in the maintenance and functioning of oligodendrocyte progenitor cells (OPCs) and post-mitotic oligodendrocytes (OLs), respectively. Crawford et al. (2010) showed that diarylpropionitrile (DPN), a synthetic and non-steroidal ERβ agonist, improves the generation of OLs and remyelination of the corpus callosum and spinal cord of mice with experimental autoimmune encephalomyelitis (EAE), a model for multiple sclerosis, even restoring nerve conduction and refractoriness of callosal axons. DPN induces a marked improvement in the number of mature OLs, myelin density and axonal conduction, leading to functional recovery in EAE mice (Khalaj et al., 2016). Also, the spinal cord of EAE mice treated with DPN display increased activation of Akt/mammalian target of rapamycin (mTOR) axis, which is not observed in a cohort of EAE mice with conditional deletion of ERβ (Khalaj et al., 2016). This finding correlates with those described by Kumar et al., who demonstrated that ERβ is crucial to enhance OLs production, myelin protein synthesis, remyelination, and axon conduction in EAE mice through the activation of the PI3K/Akt/mTOR signaling pathway, without disturbing inflammation in the corpus callosum or spinal cord (Kumar et al., 2013; Figure 2).

A growing body of evidence suggests estrogens are relevant in oligodendrogenesis by promoting oligodendrocyte differentiation in a GPER-1-dependent manner. In this regard, it has been shown that the pharmacological treatment with the classical estrogen receptor antagonist Fulvestrant (ICI-182,780) modifies the proliferative activity of NSPCs without affecting the differentiation toward the oligodendroglial lineage (Okada et al., 2010). Notably, the use of a cell-impermeable E2, a bovine serum albumin-conjugated E2 (E2-BSA), rapidly activates ERK1/2-dependent pathways, which is not inhibited by ICI-182,780 (Okada et al., 2010). Furthermore, E2-BSA does not promote proliferation of NSPCs, but it does mimic the increase in the generation of oligodendrocytes, strongly suggesting that the oligodendroglial generation from NSPCs is likely to be stimulated via membrane-associated ERs (Okada et al., 2010). In this regard, it has been shown that tamoxifen, a type of selective estrogen receptor modulator (SERM) that acts as a competitive antagonist or as a partial agonist depending on the cell type, favors the repair of demyelinated lesions in the CNS by increasing the mRNA levels of myelin basic protein (MBP) and 2′, 3′-Cyclic nucleotide 3′-phosphodiesterase (CNP), two genes highly expressed during oligodendroglial differentiation (Gonzalez et al., 2016). Furthermore, the effects of tamoxifen were blocked when a pan-antagonist was added to the media, thus supporting that tamoxifen acts as an estrogen receptor agonist in OPC differentiation. Interestingly, this tamoxifen-induced differentiation occurs in a PKCα signaling independent-manner, and not only by activation of classical estrogen receptors but also by activation of GPER-1 (Gonzalez et al., 2016). These observations correlate with those reported by Barrat et al., showing that tamoxifen-derived metabolites (i.e., 4-hydroxy-tamoxifen and endoxifen) also promote oligodendrogenesis in an estrogen receptor-dependent manner, including non-classical ERs (Barratt et al., 2016; Table 1).

### Phytoestrogens and Neurogenesis

Phytoestrogens have been previously described by their ability to promote the differentiation of diverse types of stem cells, including NSPCs (Heim et al., 2004; Oberbauer et al., 2013). *In vitro* experiments performed on embryonic forebrain-derived NSPCs revealed that hops (*Humulus lupulus* L.)-derived prenylflavonoids—the most potent type of phytoestrogens reported so far—can induce neurite outgrowth of dorsal root ganglion neurons and neuronal differentiation of NSPCs, measured by the activation of the promoter of neuronal fate-specific doublecortin (DCX) gene (Oberbauer et al., 2013). Likewise, resveratrol (3,5,4′-trihydroxy-trans-stilbene), a polyphenolic compound found in grapes and wine that acts as a phytoestrogen (Gehm et al., 1997), can regulate the proliferation and neuronal differentiation of embryonic and adult NSPCs and human bone marrow-derived mesenchymal stem cells through the activation of protein kinase A (PKA)/glycogen synthase kinase 3 β (GSK3β)/β-Catenin and PKA/ERK1/2 signaling pathways, as well as p38 kinases, sirtuin-1 (SIRT1) and AMP-activated protein kinase (AMPK) (Figure 3; Taupin and Gage, 2002; Park et al., 2012; Kumar et al., 2016; Jahan et al., 2018).

Administration of resveratrol (20 mg/kg body weight) to adult rats significantly increases the number of proliferating and newly generated cells in the hippocampus, with upregulation of p-CREB and SIRT1 proteins (Kumar et al., 2016). In contrast, experiments performed in mice showed that resveratrol (10 mg/Kg body weight) reduced the proliferation of NSPCs in the hippocampus (Park et al., 2012). *In vitro*, resveratrol exerts biphasic effects on rat-derived NPCs; low concentrations (10 μM) stimulate cell proliferation through increased phosphorylation of ERK1/2 and p38 kinases, whereas high concentrations (>20 μM) exhibit inhibitory effects (Kumar et al., 2016).

#### Xenoestrogens (Bisphenol-A) and Neurogenesis

Xenoestrogens are considered endocrine disruptors commonly associated with harmful effects in the CNS, affecting several cellular functions. Growing evidence indicates that bisphenol-A can modify the neurogenic process, affecting the proliferation/differentiation balance of NSPCs and neuronal maturation.

##### Bisphenol-A and the Proliferation/Differentiation Balance of NSPCs

Gestational exposure to low doses of bisphenol-A (20 ng/kg) change neuron distribution across cortical layers. A single injection of 5-bromo-2′-deoxyuridine (BrdU) at E14.5 showed a decrease in the number of proliferative (BrdU-positive) cells of the IV cortical layer, whereas layers V and VI showed more BrdU-labeled cells in bisphenol-A-treated than control mice (Nakamura et al., 2007).

Experiments performed by Tiwari et al. (2015b) showed that bisphenol-A has a dual role on NSPCs proliferation. Treatment of neurospheres derived from rat hippocampus with bisphenol-A at doses lower than 100 μM showed a significant increase in proliferation and cell viability. However, doses of bisphenol-A higher than 100 μM induce a substantial reduction the viability and the number of proliferating NSPCs in neurospheres, as well as the number of neurons and astroglial cells derived from them (Tiwari et al., 2015b). Similar results have been found *in vivo*, doses of 4 μg/kg body weight of bisphenol-A enhanced the number of BrdU + cells in the rat hippocampus, whereas doses of 40 and 400 μg/kg significantly reduce them (Tiwari et al., 2015b). Interestingly, most of the bisphenol-A-dependent detrimental effects on hippocampal neurogenesis are ameliorated or prevented by curcumin treatment (Tiwari et al., 2016). On the other hand, bisphenol-A decreases the number and size of oligodendroglial-committed neurospheres. Indeed, both *in vitro* and *in vivo* findings reveal that bisphenol-A alters the proliferation and differentiation of OPCs, and reduces the expression of myelination-related genes (Tiwari et al., 2015a).

High concentrations of bisphenol-A also reduce the number of neurons and significantly increases the number of glial cells located in the rat DG, suggesting a pro-glial differentiation effect on proliferating NSPCs (Tiwari et al., 2015b). Bisphenol-A treatment also reduces the number of differentiating neurons from an immortalized human NSPC line known as ReNcell VM (Fujiwara et al., 2018).

Recent investigations have demonstrated that bisphenol-A affects mitochondrial biology in hippocampal NSPCs, thus leading to increased oxidative stress and apoptosis (Agarwal et al., 2016; Figure 3). The dynamin-related protein (Drp-1) and its mitochondrial adaptor fission protein 1 (Fis-1) are the main responsible for mitochondrial fission, a process positively related to mitochondrial fragmentation and apoptosis (Frank et al., 2001; Lee et al., 2004). On the other hand, the mitochondrial dynamin-like GTPase (Opa-1) and mitofusin 1 and 2 (Mfn-1/2) are the pro-fusion proteins responsible for maintaining mitochondrial circuitry functions. Bisphenol-A treatments upregulate the levels of Drp-1 in NSPCs *in vitro* and in the rat hippocampus, leading to decreased elongated mitochondria, abnormal cristae depletion, apoptosis, and consequently loss of NSPCs proliferation/differentiation balance. In parallel, bisphenol-A reduces mitochondrial membrane potential and ATP (Agarwal et al., 2016). Bisphenol-A also decreases the levels of superoxide dismutase (SOD) and catalase, enhancing the cellular levels of ROS. In that sense, genetic and pharmacological inhibition of Drp-1 reduces all these bisphenol-A-mediated inhibitory effects, indicating that impaired mitochondrial dynamics and cytotoxicity are Drp-1 dependent (Agarwal et al., 2016). Additionally, bisphenol-A’s inhibitory effects are also diminished by pharmacological treatment with the antioxidant N-acetyl-cysteine (NAC), which similarly reduces Drp-1 levels in treated NSPCs cultures (Agarwal et al., 2016). Similarly, bisphenol-A provoked an accumulation of ROS levels in HT-22 cells, an immortalized mouse hippocampal cell line (Pang et al., 2019).

Recent investigations have shown that some of the effects of bisphenol-A in neurogenesis are mediated by the suppression of the Wnt/β-catenin signaling pathway (Tiwari et al., 2015b, 2016). In that sense, bisphenol-A promotes the expression of extrinsic Wnt inhibitory molecules (Dkk-1 and Wif-1) and decrease Wnt-3 protein levels. Bisphenol-A treatment induces the expression of the regulator GSK-3β (glycogen synthase kinase 3 β) in the hippocampus and SVZ, consequently reducing the ratio of phospho-GSK-3β/GSK-3β and enhancing the phospho-β-catenin/β-catenin one. This process prevents β-catenin nuclear translocation and its interaction with nuclear (TCF/LEF) transcription factors (Figure 3; Tiwari et al., 2015b).

##### Bisphenol-A and Neuronal Maturation

It has been demonstrated that bisphenol-A not only affects the proliferation/differentiation balance of NSPCs but also the maturation of newborn neurons. Bisphenol-A treatment (200 μM for 5–7 days) of primary rat neuron cultures showed an increase of oxidative stress and impairment of neurite outgrowth (Cho et al., 2018). This treatment also enhanced the intensity of DCX, a characteristic immature neuronal marker, indicating an inhibition or delay in the process of neuronal maturation (Cho et al., 2018). Interestingly, thicker and longer neurites appear to be formed with bisphenol-A 100 μM treatment (Kim et al., 2009). Other studies have shown that bisphenol-A blocks steroid induction of the dendritic spines in the hippocampus (CA1) and in the medial prefrontal cortex (mPFC) of rats (MacLusky et al., 2005; Bowman et al., 2015) and primates (Leranth et al., 2008). These effects consequently inhibit synaptogenesis (MacLusky et al., 2005; Leranth et al., 2008; Bowman et al., 2015) and impair spatial memory in object placement tests (Diaz Weinstein et al., 2013).

Recent studies suggest that bisphenol-A can affect the migration and maturation of cortical inhibitory GABAergic interneurons. In this context, it has been shown that bisphenol-A can alter the perinatal chloride shift, a critical period during development where the cytosolic concentration of chloride downs from ∼100 to ∼20 mM (Fiumelli and Woodin, 2007). A key element for the chloride shift is the upregulation of the chloride-extruding transporter molecule (KCC2) (Rivera et al., 1999). High neuronal chloride is essential for interneuron precursors migration from ganglionic eminences to the dorsal region of the telencephalon (also known as pallium). Once in the cortex, upregulation of KCC2 is necessary for the reduction of interneuron motility through the drop of neuronal chloride, a process which is also a precondition for the inhibitory effects of the neurotransmitter GABA and glycine in the CNS (Bortone and Polleux, 2009). A recent investigation has shown that treatments of organotypic cultures with bisphenol-A enhanced the repression of the KCC2 transcription, inducing an increment in the average speed and number of migrating neurons leaving the ganglionic eminence (Djankpa et al., 2019). In that sense, bisphenol-A exposure during a sensitive time-point of neurodevelopment could disrupt the cytoarchitecture of the cortex by over-migration of interneurons (Yeo et al., 2013). Additionally, neural repression of the KCC2 could affect synaptic maturation, compromising the function of inhibitory cortical neurons (Yeo et al., 2013).

## Conclusion and Future Perspectives

The beneficial or detrimental effects of estrogens on NSPCs biology and neurogenesis, and their effects on neuronal function and survival represent a challenging research field. The data discussed here summarize some of the roles of endogenous estrogens, estrogen-based therapies, and estrogen-like molecules on brain homeostasis, particularly in neurogenesis, gliogenesis, and regulation of the neuroprotection/neurodegeneration balance. The neuroprotective role of estrogens implies a close regulation of processes such as (i) anti-oxidant activity (Kumar et al., 2010), (ii) DNA repair (Bethea et al., 2016), (iii) synthesis of growth factors (Scharfman and MacLusky, 2006), (iv) synaptic plasticity (McEwen and Milner, 2007), and (v) the blood flow to the brain (Chambliss et al., 2000). These mechanisms are vital for maintaining neuronal survival and counteract the increased risk of cognitive decline and neurodegenerative disorders associated with aging (Zhao and Brinton, 2006; Engler-Chiurazzi et al., 2017). In fact, women with premature menopause who do not receive estrogen treatment show an increased risk of developing Alzheimer’s disease (Shuster et al., 2010); while epidemiological reports support that HRT acts as a neuroprotective factor in post-menopausal women (Song et al., 2020). The study of molecular and cellular mechanisms underlying estrogen-mediated neuroprotection represents a field of research with exciting projections since its impact transcends to a series of processes that regulate nervous tissue homeostasis. Therefore, phytoestrogens and their metabolites, which have been shown to replicate the benefits of endogenous estrogens, and estrogen-based therapies, are considered promising approaches for preventing the onset and progression of neurodegenerative disorders. By contrast, xenoestrogens commonly found in polymers of plastic polycarbonates, such as mineral water bottles and lacquer coating in food cans, can act as endocrine disruptors that significantly alter estrogen-mediated signaling pathways (Brotons et al., 1995; Wagner and Oehlmann, 2009). This is key since bisphenol-A molecules are joined by ester bonds, which are disrupted by high temperatures or pH chances, and subsequently released on water and food (Brotons et al., 1995; Wagner and Oehlmann, 2009), thus tempting to speculate on the consequences of chronic exposure to these molecules, especially in post-menopausal women.

The published data show that estrogen-like molecules can mimic or block endogenous estrogen’s biological effects through a series of non-genomic responses (Park et al., 2012; Kumar et al., 2016; Jahan et al., 2018). Phytoestrogens are mostly beneficial to the processes of proliferation and differentiation of NSPCs; however, some detrimental effects have also been reported. A possible explanation for this dichotomy could be given by diverse factors, such as the volume of consumption, time of exposure, genetic background, and degree of development. It is essential to consider that most *in vitro* studies have evaluated the effects of phytoestrogens in an isolated manner, leaving aside the fact that a set of molecules induces the effects, and not only a single one. Also, an important part of their activity can be attributed to the generation of diverse microbiome-derived metabolites (Setchell et al., 2002; Branca and Lorenzetti, 2005), some of which are not generated by the entire population (Setchell et al., 2002; Setchell and Lydeking-Olsen, 2003; Legette et al., 2014). So, it is expected that a similar set and quantity of phytoestrogens can induce dichotomic effects depending on the individual’s idiosyncrasy. Besides, the short half-life prevents bioaccumulation and may restrict their activity in a given period (Yang et al., 2012; Legette et al., 2014), mimicking endogenous estrogens.

Endocrine-disrupting chemicals such as bisphenol-A mostly induce detrimental effects in NSPCs, reducing their self-renewal ability and cell potency. Although the potential human health risks associated with these chemicals represent a significant public health concern, some controversy has been generated by their relatively low biological potency and affinity for classical and non-classical ERs (Rider et al., 2010). In contrast to phytoestrogens, these chemicals tend to bioaccumulate and induce persistent and refractory effects, even though their environmental concentrations oscillate in a relatively low range (Liu et al., 2012). Clinical and preclinical evidence supports the fact that the maternal transfer of bisphenol-A can induce long-term effects in diverse systems, including the CNS. Actually, exposure to bisphenol-A during gestation has been shown to produce glucose intolerance, insulin resistance, and a reduction in the number of pancreatic β-cells (Alonso-Magdalena et al., 2010). In the same direction, it has been reported that bisphenol-A generates apoptosis in some cerebral nuclei and variations in the expression of ERs in others (Kubo et al., 2001; Rebuli et al., 2014), leading to consider its potential for inducing changes in the cellularity and/or functionality of diverse areas of the brain or even reduce the sensitivity of others to endogenous estrogen. Both *in vitro* and *in vivo* studies reveal that bisphenol-A exposure during postnatal development alters ER phosphorylation and its translocation to the nucleus in the hippocampus (Xu et al., 2014). Additional studies indicate that bisphenol-A affects estrogen signaling and induces severe alterations in the proliferation and differentiation processes of NSPCs (Tiwari et al., 2016). These chemicals can induce modifications in the brain’s function by disrupting molecular and cellular mechanisms associated with neurogenesis, gliogenesis, neuronal survival, and brain repair.

The data mentioned above consider several findings in both embryonic and adult NSPCs, as well as OPCs. It is noteworthy that the CNS-residing NSPCs show many biochemical and morphological differences throughout life, but the origin of adult NSPCs -type B cells- has been traced from early neurodevelopmental stages (Merkle et al., 2004). Elegant imaging studies have proven that radial glial cells can divide asymmetrically to generate type B cells that remain quiescent until they are reactivated in adulthood, without affecting the prenatal neurogenesis; a process that highlights the efficiency of the mechanisms of cellular specification (Merkle et al., 2004; Kriegstein and Alvarez-Buylla, 2009; Kheirbek, 2015). Also, the origin of OPCs is traced early in the formation of the mammalian CNS and comprises multiple and sequential waves of production of OPCs that end in colonization and positioning in the cortex and spinal cord (Kessaris et al., 2006; Kriegstein and Alvarez-Buylla, 2009). Exposure to these types of endocrine chemical disruptors during pregnancy could alter the proliferative activity and cell potency in both populations of NSPCs, but their effects could be reflected only later in life. Future research focusing on the importance of these chemicals as factors that predispose to developing diverse neurological conditions or even their impact on ongoing illness is needed.

## Author Contributions

FB-B and MM-R: drafting original manuscript. PL-C, LM, CF, and LB: reviewing and editing. LM, CF, and LB: validation. AO, FR, CF, and LB: supervision and funding acquisition. All authors contributed to the article and approved the submitted version.

## Conflict of Interest

FB-B was employed by company Cells for Cells (Chile). The remaining authors declare that the research was conducted in the absence of any commercial or financial relationships that could be construed as a potential conflict of interest.
